# Diagnosis and Therapy of Tuberculosis of the Mesenteric Glands of Children

**Published:** 1905-11

**Authors:** 


### Diagnosis and Therapy of Tuberculosis of the Mesenteric Glands of Children.

Hecht, (*Therapie d. Genewart,* No. 4, 1905) writes: The diagnosis can be made with certainty only when the affected glands or their conglomerates are palpable. With early, rational and persistant treatment the prognosis is by no means generally unfavorable. Besides the warm compresses and green soap inunctions recommended by others, he has used creosotal in this localisation of scrofulosis, with the same success which he recorded in the homologous swellings of the bronchial and cervical glands (see same journal, p. 334, 1904.)

